# Population dynamics of *Hippophae rhamnoides* shrub in response of sea-level rise and insect outbreaks

**DOI:** 10.1371/journal.pone.0233011

**Published:** 2020-05-21

**Authors:** Mathieu Decuyper, Robbert van den Dool, Pieter A. Slim, A. T. (Loek) Kuiters, Jeroen M. Jansen, Ute Sass-Klaassen

**Affiliations:** 1 Wageningen Environmental Research, Wageningen University & Research, Wageningen, The Netherlands; 2 Laboratory of Geo-Information Science and Remote Sensing, Wageningen University and Research, Wageningen, The Netherlands; 3 World Agroforestry (ICRAF), Nairobi, Kenya; 4 Centre for Crop Systems Analysis, Wageningen University and Research, Wageningen, The Netherlands; 5 Nederlandse Aardolie Maatschappij BV, Assen, The Netherlands; 6 Forest Ecology and Forest Management Group, Wageningen University and Research, Wageningen, The Netherlands; Estacion Experimental de Zonas Aridas, SPAIN

## Abstract

The coastal vegetation of islands is expected to be affected by future sea-level rise and other anthropogenic impacts. The biodiverse coastal vegetation on the eastern part of the Dutch Wadden Island of Ameland has experienced land subsidence caused by gas extraction since 1986. This subsidence mimics future sea-level rising through increased flooding and raising groundwater levels. We studied the effects of this relative sea-level rise and other environmental factors (i.e. insect outbreaks, temperature and precipitation) on the population dynamics (i.e. cover and age structure and annual growth) of the shrub sea-buckthorn (*Hippophae rhamnoides* L.) in young (formed after 1950) and old (formed before 1950) dune areas over a period of 56 years (1959–2015). We found an increase in sea-buckthorn cover in the young dune areas since 1959, while over time the population in the old dunes decreased due to successional replacement by other species. With the increasing age of the young dunes, we found also a decrease in sea-buckthorn after 2009. However the sharp decrease indicated that other environmental factors were also involved. The most important determinant of annual shrub growth appeared to be five outbreaks of the brown-tail moth (*Euproctis chrysorrhoea* L.), in the last decade. Relative sea-level rise caused more frequent flooding and reduced growth at lower elevations due to inundation or soil water saturation. This study clearly indicates that sea-buckthorn is affected by relative sea-level rise, but that other ecological events better explain its variation in growth. Although shrub distribution and growth can be monitored with robust methods, future predictions of vegetation dynamics are complicated by unpredictable extreme events caused by (a)biotic stressors such as insect outbreaks.

## Introduction

Due to global warming, sea levels are rising and are predicted to have increased by 90 cm by the end of this century according to the IPCC scenario RCP8.5 [1]. This poses a global threat to shorelines due to coastal erosion and increased storm flooding, which induce indirect effects such as loss of biodiversity of coastal vegetation [2]. Small islands with (semi-) natural shorelines are especially vulnerable [3]. This is the case for the UNESCO protected Wadden Sea and its barrier islands. One of these islands is Ameland, a biodiversity hotspot that stands as a model for coastal dunes of the Wadden Sea area of the Netherlands. Harbouring many of the protected habitats, bird species, and red list flora and fauna species typical of coastal dunes, this dynamic area is of great importance for nature conservation [4,5]. However, the island is also an area where natural gas has been extracted since 1986; a reason for environmental concern in the Netherlands. Compaction of gas-reservoir rock causes soil subsidence at the earth’s surface. Maximum subsidence on the island was predicted to reach approximately 40 cm by 2020 [6]. This subsidence could influence the increase in the relative seawater level and groundwater level which could affect the coastal vegetation. The consequences of this gradual soil subsidence are comparable with the expected sea-level rise of 52–98 cm by 2100 (IPCC) [1]. This is why Ameland can serve as model island to make predictions on the impacts of sea-level rise in islands with similar dune slack and salt marsh habitats.

Effects of flooding and increased groundwater level on vegetation can include root damage due to hypoxia, build-up of toxic compounds and increased susceptibility to pathogens [7]. Prolonged flooding can also cause mortality from asphyxiation or post inundation stress. Plant tolerance levels to flooding are can be influenced by calcium levels, age, disease and other factors lowering vitality [7].

Besides the effects of flooding on vegetation, the positioning in the dunes also influences plant communities. Plants at high elevation dunes rely on soil moisture reserves and their own specific adaptations to prevent damage and mortality from drought [8,9]. As dunes mature, calcium levels gradually drop through leaching, reducing the plants’ resistance to disease [10]. In older dunes the higher presence of root-feeding nematodes [11, 12] makes the soil more suitable for taller shrub and tree species in later successional stages due to an accumulation of soil organic matter [13]. Other biotic and abiotic factors, in addition to flooding can impact coastal plant communities. High temperature and salt spray can affect plant growth and population dynamics [14], and insects can cause defoliation of plants which impacts their growth. Recently outbreaks of brown-tail moths (*Euproctis chrysorrhoea* L.) have caused complete defoliation of some plant species. These insect outbreaks could become more frequent with climate change [15]. While having largely disappeared on the mainland, this larval overwintering species still has a major presence in the coastal regions of the Netherlands [16].

**Fig 1:**
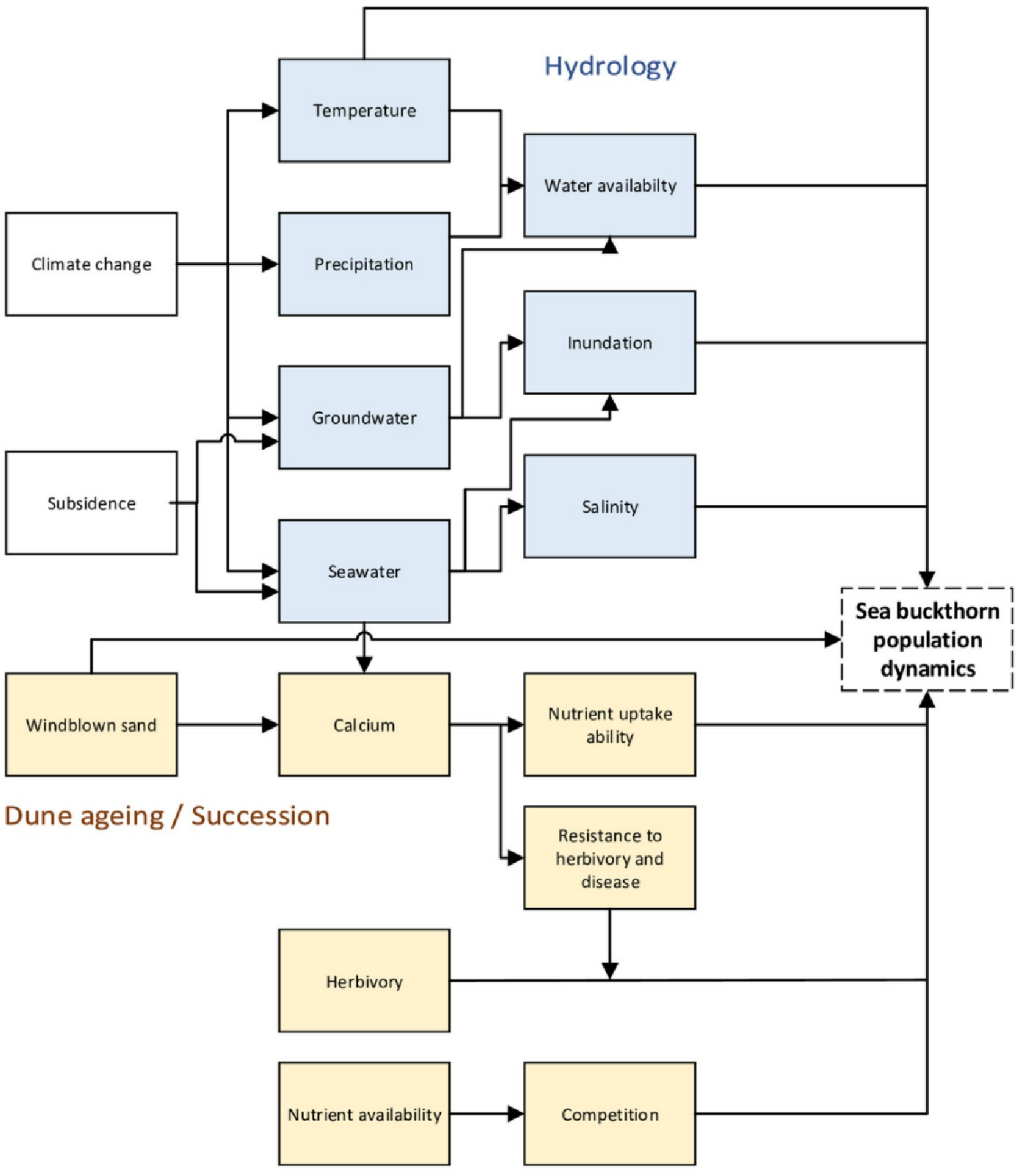
Conceptual diagram showing the factors directly or indirectly influencing the population dynamics of sea-buckthorn, which can be grouped in hydrology and dune ageing related factors.

In this study we assess the effect of relative sea-level rise on the population dynamics and growth of sea-buckthorn (*Hippophae rhamnoides* L.), a common plant species inhabiting the study area. While areas designated under Natura 2000 as the habitat type ‘Dunes with *Hippophae rhamnoides* (H2160)’ are protected, expansion of this habitat type will be at the cost of more vulnerable habitat types such as ‘Humid dune slacks (H2190)’ in low situated areas and ‘Grey dunes (H2130)’ in high situated areas [5,17,18]. Over the last decades, shrub encroachment by sea-buckthorn has been observed near the fore dunes, as well as in newly formed dunes [19]. However, one study also showed increased shrub mortality, possibly due to extreme floods and insect outbreaks in specific years [20]. Sea-buckthorn is known to have a medium tolerance for flooding by relying on adventitious roots to counter the effects of hypoxia [21]. Tolerance to flooding and salinity depends on the plant life stages, e.g. seeds may fail to germinate and seedlings show reduced shoot growth with increasing salinity [22]. Exact tolerance levels of sea-buckthorn to flooding is however unknown. The higher presence of root-feeding nematodes can partially explain the decline of sea-buckthorn in older dunes [19]. Older dunes with an accumulation of soil organic matter are more suitable for taller shrub and tree species in later successional stages [13], and these species will outcompete the shade intolerant sea-buckthorn. Insect outbreaks can have negative effects on sea-buckthorn, including the brown tail moths. An overview of the factors influencing the population dynamics of sea-buckthorn can be found in Fig 1.

In this paper we use dendrochronology and aerial photographs to answer the following research questions: How did the population of sea-buckthorn on Ameland develop during decades of relative sea-level rise? What are the main drivers of its development? What are the main factors influencing sea-buckthorn growth, and what could be the effect of soil subsidence on growth? We test the hypothesis that population dynamics of sea-buckthorn in the area is positively affected by the formation of new (young) dunes, but over time, negatively affected by ageing of dunes, insect outbreaks and increased flooding with seawater related to soil subsidence.

## Materials and methods

The field research included the collection of plant material. The field permit (number: BIG/2015/5640) was granted by It Fryske Gea, regional society for nature conservation.

### Study species

**Fig 2:**
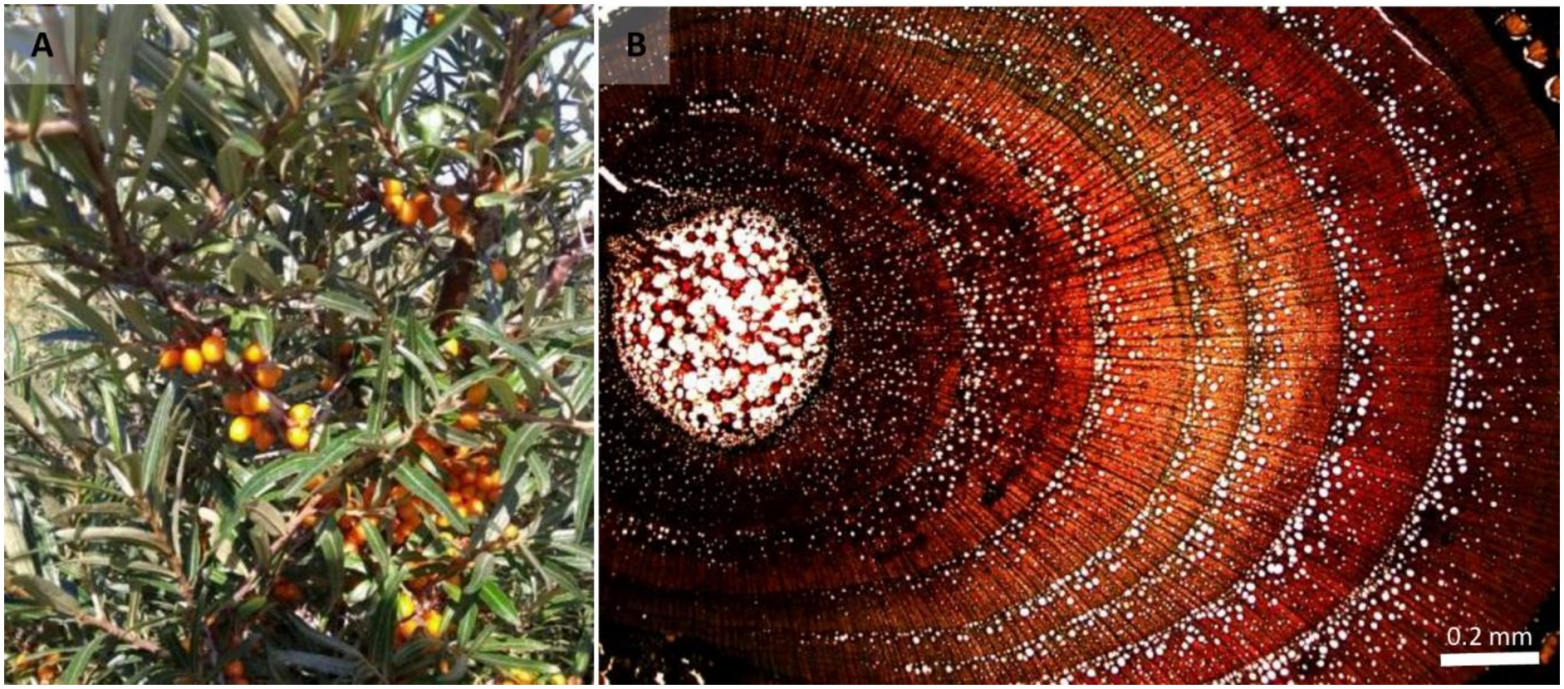
A) Sea-buckthorn (*Hippophae rhamnoides* L.) in the growing season. B) Micro-thin section of the stem indicating the tree-rings.

Sea-buckthorn (*Hippophae rhamnoides* L.) is a deciduous, dioecious shrub. It is often the first woody plant establishing in natural coastal dune succession [23] (Fig 2A). While the environmental conditions are too harsh for the species in the Marram grass (*Ammophila arenaria* L.) dominated ‘White dunes’, the shrub is able to settle under less dynamic circumstances with its bird dispersed seeds. Sea-buckthorn is a nitrogen fixing species that proliferates under calcium rich conditions, grows up to half a meter in height annually and shows strong lateral vegetative growth, sprouting root suckers vigorously [24]. It is known to be resistant to stress caused by cold, heat, drought and, to some extent, inundation and the associated salinity [24].

### Study area and sampling

**Fig 3:**
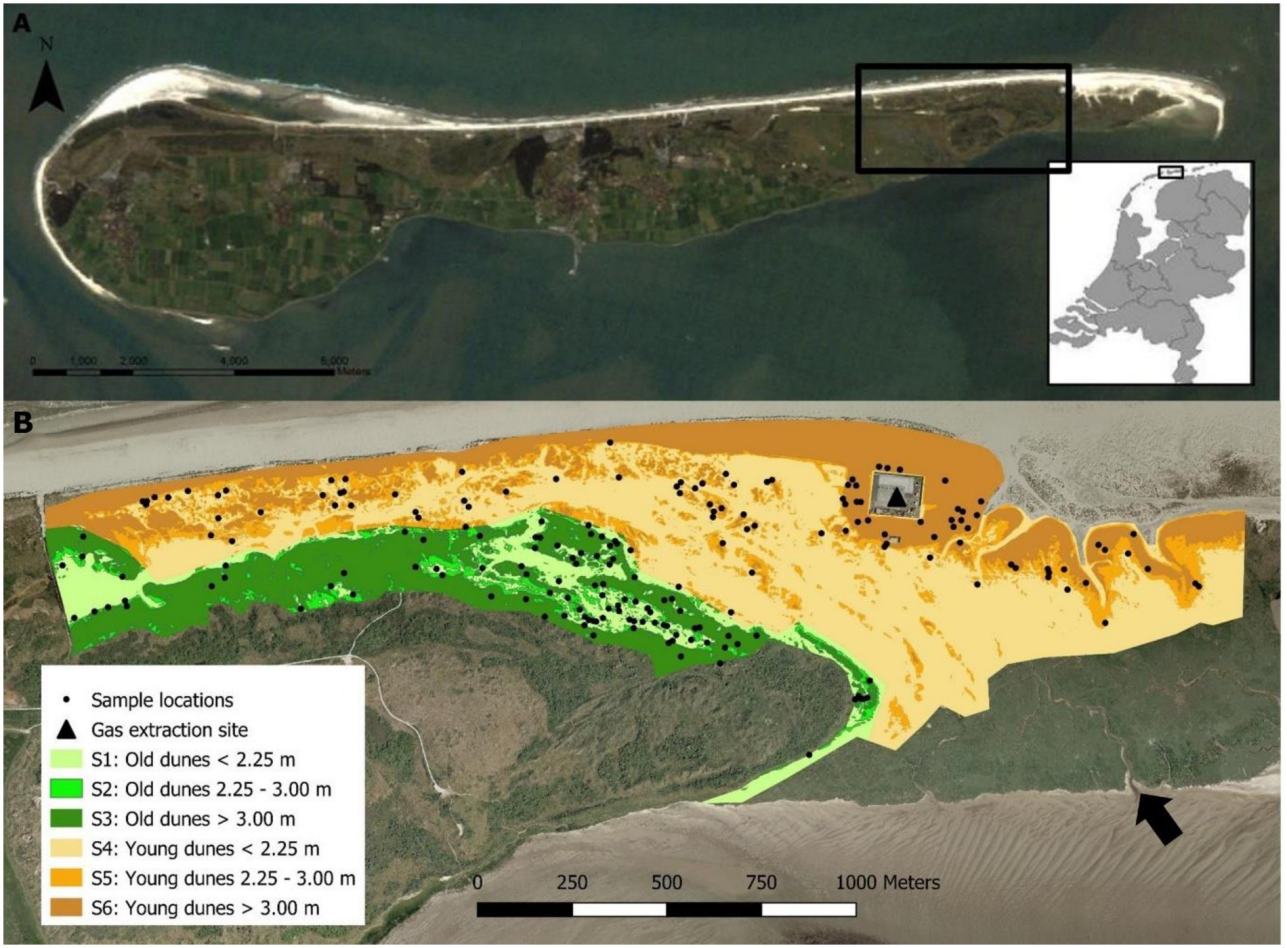
A) Location of the Wadden Sea Island of Ameland in the northern part of the Netherlands with the delineation of the study area on the eastern end of Ameland (black rectangle). Map source: USGS Landsat image. B) The study area with indication of its different strata, the gas extraction site (triangle), sample locations (dots) and seawater inflow (arrow). Map source: Topographic service Cadastre.

The study was carried out in the eastern part of the Wadden Island of Ameland (53°27'43"N, 5°54'12"E) (Fig 3A). Ameland has been part of the UNESCO word heritage site Wadden Sea since 2009 and is designated as a Natura 2000 site because of its ecological value. An open connection with the Wadden Sea in the southeast allows seawater to flow into the area during winter storms, inundating salt marshes and dune slacks for several months (October-April) [25]. This creates gradients in salinity that match gradients in soil moisture, calcium availability and nutrient richness [26]. Within this mosaic of gradients in environmental factors that influence species performance, competition and predation codetermine the success of individual species or species assemblages. This results in a diverse coastal dune ecosystem.

The study site was chosen within an area with soil subsidence, consisting of dry dune grasslands, moist dune slacks, and dunes with sea-buckthorn. Since the start of gas extraction in 1986, subsidence up to approximately 40 cm in the middle of the subsidence area with a diameter of 12 km occurred by 2017 [6]. To capture the difference in environmental conditions, the study area was stratified by elevation and dune age. Three elevation classes were determined based on the frequency of seawater flooding [25]: areas below 2.25 m NAP (Amsterdam Ordnance Datum) are flooded on a yearly basis; areas between 2.25 m and 3.00 m are flooded roughly every other year; areas higher than 3.00 m are almost never flooded. The classification into strata was based on the AHN2 elevation model of the area with a spatial resolution of 0.5 m from 2008 (https://www.ahn.nl/). Furthermore, two dune age classes were determined based on periods of dune formation described by Wiertz (1990) [27]. For the purpose of this study, dunes formed before 1950 are considered ‘old dunes’ and dunes formed after 1950 are considered ‘young dunes’. Most of the ‘young dunes’ were formed in the period between 1959 and 1969. The dune ages, together with the elevation classes resulted in six strata indicated with “S1-6” as shown in Fig 3B.

Sampling locations of sea-buckthorn for the dendrochronological analyses were based on a classification of a recent aerial photograph indicating the current extent of the sea-buckthorn population. The original 2009 photograph was selected as it was the most recent high-colour contrast photograph available at the time this study was planned. The classified sea-buckthorn cover in each stratum was used to randomly determine a set of > 200 sample points. A minimum distance of 7 m between the points is respected, as a pilot study has shown that there is strong vegetative reproduction by *H*. *rhamnoides*. Not taking this into account would lead to pseudo-replication as nearby growing plants could be connected and hence form one organism. Per stratum, 33 points were used as sampling locations and the remaining as reserve points (Fig 3B). Sampling locations were visited in the field using RTK-GPS (Real Time Kinematic-Global Positioning System) and the closest living sea-buckthorn shrub was selected and harvested. In case a location had no sea-buckthorn within a 10 m radius, a reserve point was used.

### Dendrochronological analyses

Samples for tree-ring analyses were obtained by harvesting a stem segment at the base of the shrub. Increment cores were not a viable option due to the difficulty in determining growth rings, e.g. wedging rings, and irregular eccentric tree-ring patterns as is the case for many shrub species (e.g. Hawthorn–*Crataegus monogyna* Jacq.) [28,29]. Sea-buckthorn has a semi ring-porous wood structure with larger earlywood vessels formed in the beginning of the growing season, followed by smaller latewood vessels formed during summer (Fig 2B).

In August and September 2015, a total of 196 stem discs from sea-buckthorn shrubs were taken. The upper disc surface was manually cut with a sharp razor blade to expose the tree rings. Tree-ring widths were measured using a Lintab measuring table and TSAP-Win software (Rinntech). Up to three radii were measured when possible to improve the precision of the measurements; individual series (i.e. 33) were visually cross-dated first within a shrub and then between shrubs per stratum.

The shrub growth series were detrended to correct for age related growth trends using Regional Curve Standardization [30] in which a modified negative exponential curve was fitted through the radial growth vs age plot, and ratios for each year of each sample were determined [31] (S1 Fig). The resulting measurements no longer contain the average growth trend, but do still show individual differences and do not obscure differences between strata [30]. The detrended ring-width series of the samples within a stratum were averaged for the statistical analysis.

### Image classification from aerial photographs

To determine changes in sea-buckthorn cover percentages over time, aerial photographs (from the Dutch Topographic service Cadastre) were collected from archives (S1 Table). Photographs were panchromatic (1949–2004) or in colour (after 2009). Aerial photographs were imported in ArcGIS (ESRI Company) and, if necessary, mosaicked, geo-rectified and geo-referenced to the digital image of 2014. In total 13 overlapping aerial photographs were available of which eight were considered unsuitable (due to poor image quality), and five were suitable (1959, 1986, 2000, 2009 and 2014) to be used in the analysis (S1 Table).

Colour images were transformed to greyscale for a more equal signal-to-noise ratio between images and supervised, pixel-based image classification was applied in ArcGIS [32]. Vegetation maps and experts were consulted to determine classification training areas and visually verify the classification accuracy [33–37]. Only those areas with homogenous sea-buckthorn cover were used as training areas for the classification. Sea-buckthorn has a blueish green colour on aerial photographs, which shows up darker than other vegetation (e.g. other woody species). Some areas were manually adjusted in accordance with the vegetation maps (i.e. clipped out when other species caused misclassification).

### Environmental variables

#### Sea level

The sea level information, stored every hour (before 1987) or every 10 minutes (since 1987), was obtained from two tide stations: Nes (1975–1981, 10 km from the study site centre) and Wierumergronden (1981–2015, 3 km from the study site centre) (http://live.waterbase.nl/). Past studies on the influence of seawater in the study area were of insufficient length and spatial resolution for a comparison of sea-buckthorn growth at different elevations [25,38]. Therefore the measured elevations at sampling locations were corrected for historic levels of subsidence using the simulation model ‘AMELAND_GRIDS_2014’, a model of the gas extraction company (Nederlandse Aardolie Maatschappij BV). Seawater levels procured by this method were in line with other studies in the Wadden Sea area [39,40]. We compared actual elevation with seawater levels at the sample locations, taking soil subsidence (accuracy of +/- 2 mm) into consideration. In this way we could calculate the relative sea-level rise (long-term average sea-level rise, including the effect of subsidence) per year for each stratum. From this data, several variables such as flood frequency and the maximum height of seawater at the sample location, could be procured and averaged per stratum (S2 Fig). Seasonal seawater flooding could be derived, and averaged for each period of January until September of a given year plus November and December of the previous year (S3 Fig). Winter flooding can create unfavourable anaerobic conditions for the roots, and can affect wood formation during spring [41].

#### Groundwater

Data on groundwater depths were gathered from six piezometers (1989–2015) within the study area and a piezometer situated just outside the study area (1975–1988). Piezometer data, which consisted of roughly 6 measurements each year, were used for average groundwater depths over three periods: the whole year, the growing season (April-August) and the winter season (September-March). Groundwater depths (cm) were corrected for soil subsidence over time and values are negative in wetter years (i.e. lower depth) and positive in drier years (i.e. higher depth) (S4 Fig). Groundwater depth can indicate physiological stress endured by the shrubs due to inundation or drought. We also included the squared groundwater values as an additional variable because the regular groundwater variable can only have a positive or negative relation to growth in the regression. In this way we also take into consideration both extreme low and high groundwater levels, which we believe might have an effect on the growth of sea-buckthorn.

#### Precipitation and temperature

Monthly precipitation data was collected from the weather station on the island (KNMI—Royal Netherlands Meteorological Institute; station Nes, 1975–1995) and from the gas extraction site in the study area (1996–2015) (S5 Fig). In addition, evaporation data from multiple weather stations were gathered in order to calculate net precipitation. This represents the amount of moisture that is actually available to the vegetation [42]. Sum of net precipitation was calculated for the whole year, and for the growing season (April-August) and winter (September previous year until March) separately. Temperature data were collected from the nearest station with available temperature data, Leeuwarden (KNMI). Yearly and seasonal (growing season and winter) average temperatures were determined (S6 Fig).

#### Defoliation by brown-tail moth

**Fig 4:**
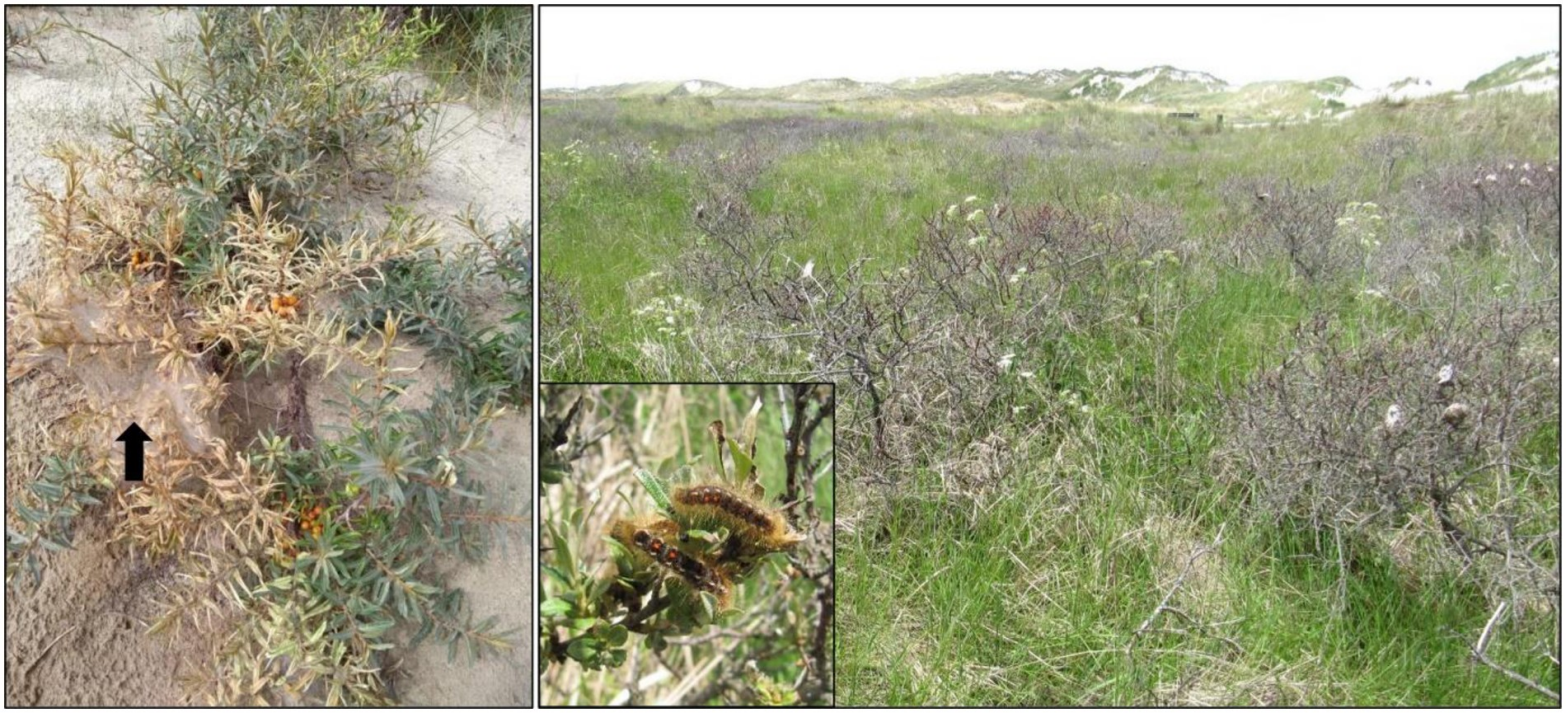
Leaf welting and defoliation by brown-tail moth (*Euproctis chrysorrhoea*). Black arrow indicates the caterpillar nest.

Brown-tail moth (*Euproctis chrysorrhoea* L.) outbreaks can have a large impact on sea-buckthorn due to defoliation-induced die-back [20,43] (Fig 4). We collected data from two sources: firstly a program monitoring damaging insect outbreaks since 1946 was used, in which the occurrence was registered through a network of volunteer green sector professionals [16]. An approximation of the occurrence of outbreak years (a binary variable) over the 1975–2010 period on Ameland could be constructed using the sum of the number of records, multiplied by the severity of the outbreak for the province of Friesland. Secondly, data from the Dutch ‘National Database Flora and Fauna’ (NDFF) with the number of sightings and number of caterpillars from the entire island of Ameland was acquired to estimate outbreaks over 2010–2015 (www.ndff.nl). For the NDFF data, years with more than 1000 observations were considered years with an insect outbreak. Years with outbreaks are: 1978–1979, 1984–1985, 1993, 1996, 2007, 2010–2011, 2014–2015.

### Statistical analysis

Statistical analyses were done using R software (version 3.2.3) [44]. Detrending of ring-width data was performed using the program CRUST [45]. Dendrochronological descriptive statistics were done with the ‘dplR’ R-package [46]. Since the young dunes (S4-6) were not fully formed by 1975, the statistical analysis for comparing the differences between strata was carried out for the period 1991–2015. One-way ANOVA was used to test differences in average growth (n = 196) between strata (n = 6) and differences in growth trends between the strata were tested with linear regression analysis.

For to the same reason, the statistical analysis on the relation between growth and environmental variables was carried out for two periods: from 1975–2015 (41 years) for the old dunes only, and from 1991–2015 (25 years) for the old and young dunes. Before analysis, explanatory variables (i.e. moth outbreaks, seawater floods, groundwater, net precipitation, temperature) were checked for multicollinearity using the ‘vifstep’ function of the ‘usdm’ R-package [47]. Variables were stepwise removed until all variables had a VIF (variance inflation factor) < 3. All independent variables (except the binary brown-tail moth outbreak variable) and the dependent growth variable were centred and standardized to allow better interpretation of model parameter estimates [48]. GLS (Generalised Least Squares) models were constructed for each stratum based on remaining variables and meaningful interactions. The factorial part of these models was the same for the analysis of each stratum; only in the analysis of the high elevation strata (S3 and S6) seawater related variables were not included. GLS models were adapted for temporal autocorrelation and normality, and independence assumptions were checked for each model using normalized residuals in histograms and residuals vs predicted and residuals vs variable plots respectively. The best explaining models were achieved using multi-model inference with the ‘dredge’ and ‘average.model’ functions of the R-package MuMIn [49], using a 2 AICc threshold [50]. Contrary to stepwise regression, the best explaining models are unbiased by the model selection process [51]. In multi-model inference, models with similar explanatory power compared to the best explaining model were not removed but used in final model construction by averaging parameter values [52].

## Results

### Vegetation cover

**Fig 5:**
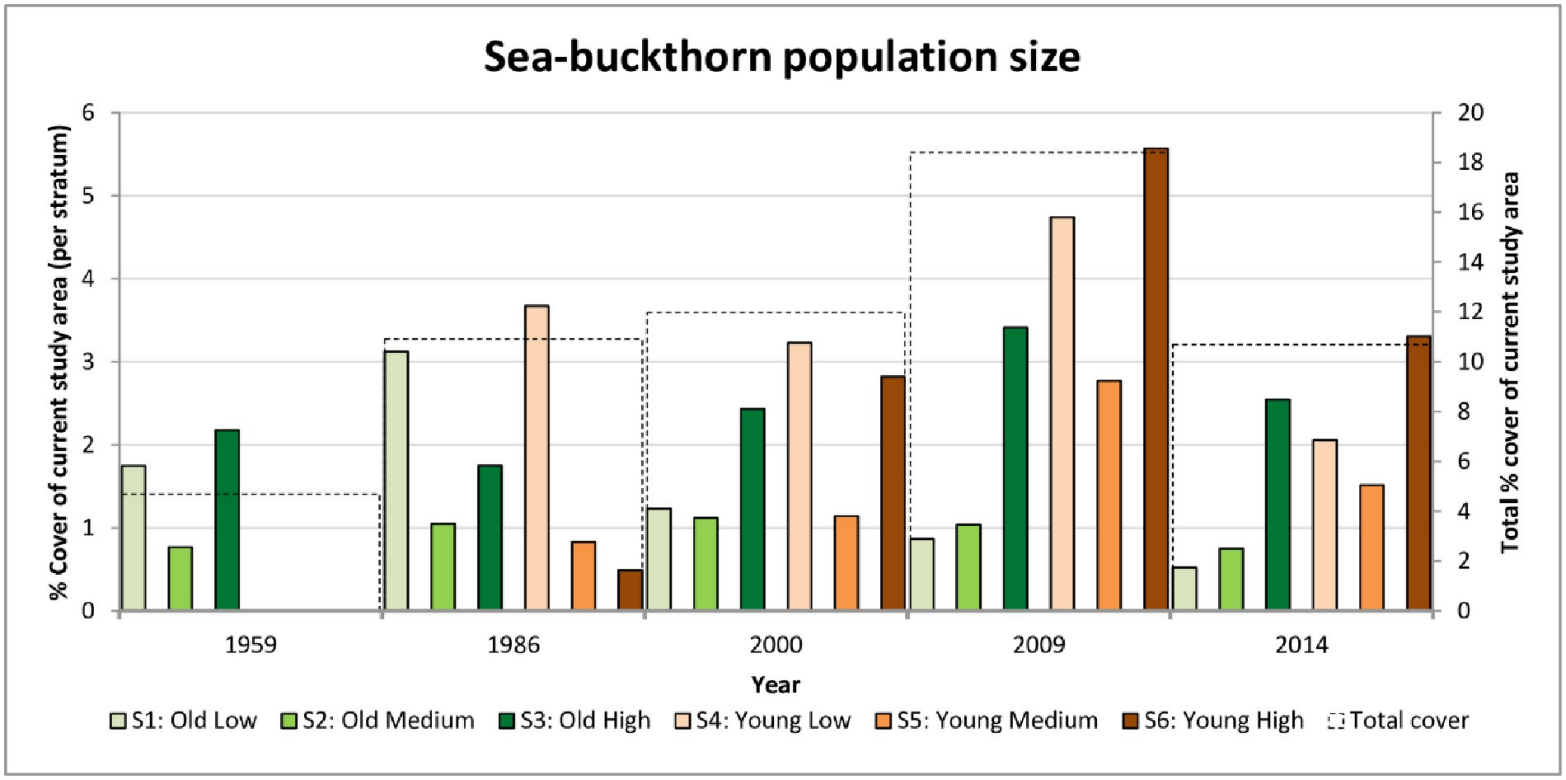
Sea-buckthorn cover (%) in the different strata for each photograph. 1 percent equals 1.5 hectares of the study area. The dotted boxes represent the sum of the population cover (%) over all strata.

Aerial photographs allowed the estimation of sea-buckthorn cover percentages in the different strata over time (S7–S11 Figs). In 1959 the young dunes were not formed yet and the old dunes had low areas not covered with scrub. In 1986, all of the current study area had developed. In the old dunes the lower areas were largely covered with sea-buckthorn (3.12%) and the population in the younger dunes was mainly limited to areas currently below 2.25 m (3.75% in comparison to 5% in the young dunes in total). Only 15 year later, in 2000, the lower areas in the old dunes show a remarkable decrease in cover (from 3.12% in 1986 to 1.23% in 2000), while the young dunes show a large increase in cover at high elevations. A large part of this increase in shrub cover was due to the artificially created dune in 1986 around the extraction site, on which a few sea-buckthorn shrubs, together with other plant species were planted. However, the majority of the increase of sea-buckthorn cover was due to natural establishment on young dunes. The 2009 classified image shows cover percentages which were higher than in previous years for most strata, especially in the young dunes. Finally, 2014 is characterized by lower cover values for all strata (Fig 5).

**Fig 6:**
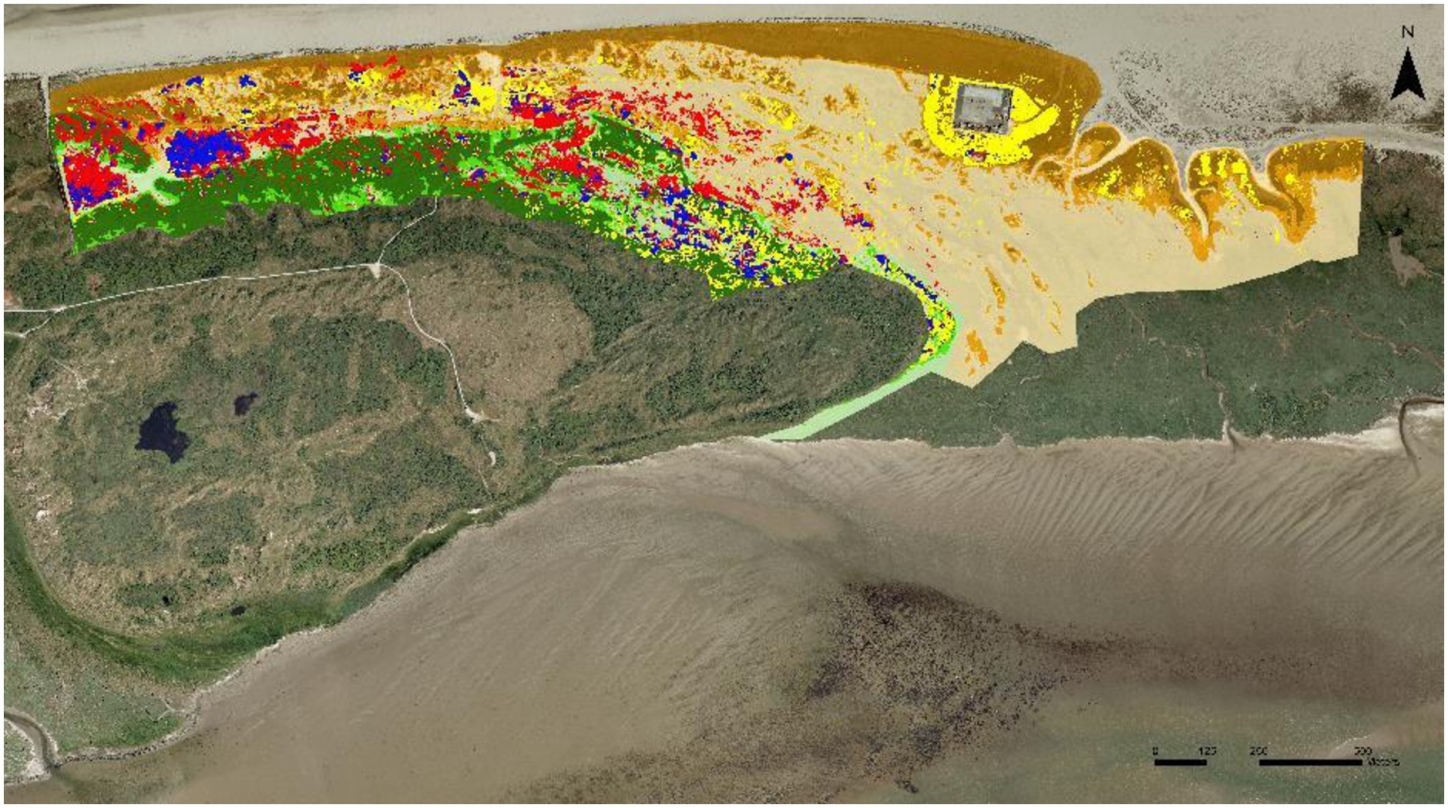
Sea-buckthorn cover changes between 1986 and 2000 (red = disappeared, yellow = appeared, blue = remained), indicating extensive mortality at low elevation in this period. The other colours represent the strata as denoted in Fig 3B. Note that the larger blue/red patches in the far west are sea-buckthorn mixed with willow species (*Salix* sp.).

A comparison between successive classified images indicated where areas of shrub expansion and mortality are located, and where the population stayed stable (Fig 6; S12–S15 Figs). Much of what was sea-buckthorn in the higher old dunes in the period before gas extraction (i.e. 1959–1986), seems to have disappeared by 2014 (total loss over all strata = 24%). This is not visible when comparing surface-area percentages alone, as new sea-buckthorn also established over the same period in the same stratum slightly further to the north (total gain over all strata = 67%).

Mortality over large areas (38%) took place in lower locations between 1986 and 2000 (red colour in Fig 6). While this decline took place in both the old and young dunes, it is not reflected in the total cover percentage of the young dunes (S4) as there was also expansion (total gain over all strata = 43%). Shrub expansion was mainly situated on the artificially created dune around the gas extraction site (some sea-buckthorn was planted and it quickly expanded). Between 2000 and 2009 further mortality (total loss over all strata = 26%) in low areas (S1 and S2) is visible in the westward slacks (S14 Fig). This was mainly due to management activities in the winter of 2005 in which scrub was removed along with the top soil. New patches of sea-buckthorn shrubs did establish in both young and old dunes (total gain = 52%). Comparison of the 2009 and 2014 photographs showed that there was mainly mortality in the young dunes (S15 Fig), with a total loss over all strata of 53%. Summing up all strata, the percentage sea-buckthorn loss was the highest between 2009 and 2014, while the gain and no change percentages were at its lowest in that period (S16 Fig).

### Shrub establishment and growth

**Fig 7:**
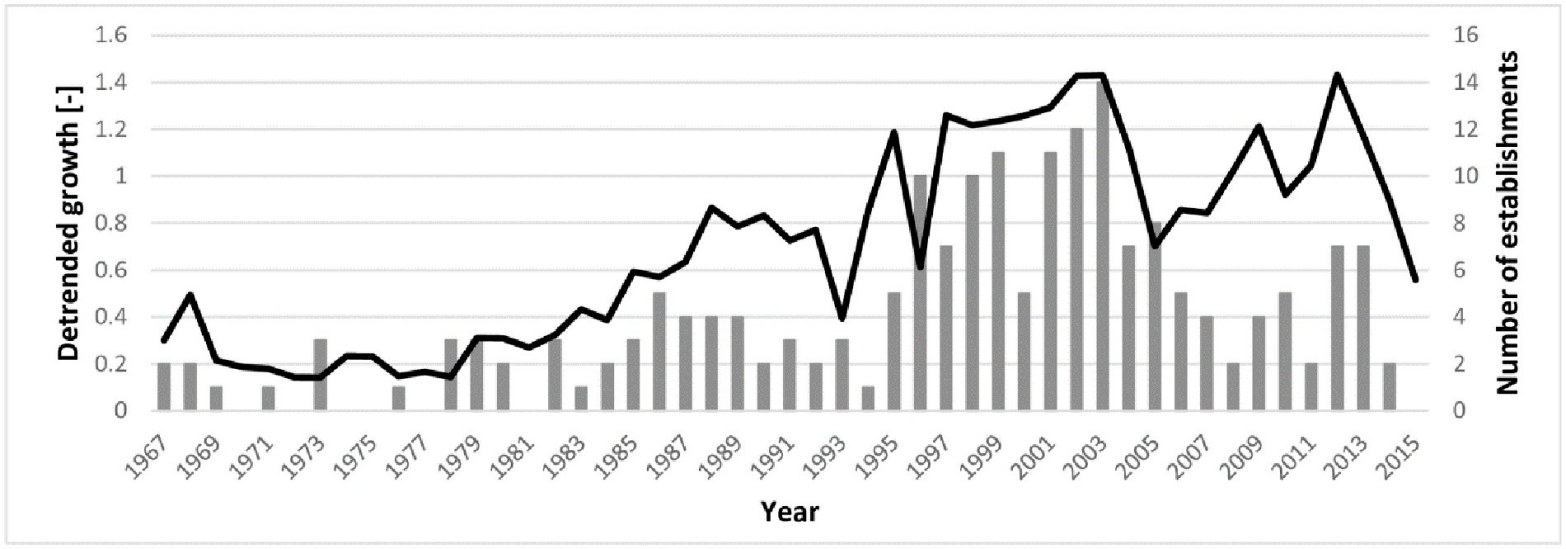
Establishments (bars) as determined through tree-ring measurements (i.e. age), and the average growth rate (line) of all samples combined (N = 196).

Average age of the sampled sea-buckthorn shrubs was 18 years, with the older samples, dating back to 1959, mainly located in the old dunes (S2 Table). Establishment was not constant and seems to have been inhibited in two periods: 1991–1996 and 2007–2015 (Fig 7; S16 Fig). The period in between (1996–2007) showed the highest rates of establishment with nearly half of the total sample size. Establishment before the 1990’s seems low, but this is probably due to mortality that took place over time (i.e. sea-buckthorn does not become extremely old, with a maximum age of 57 years).

**Fig 8:**
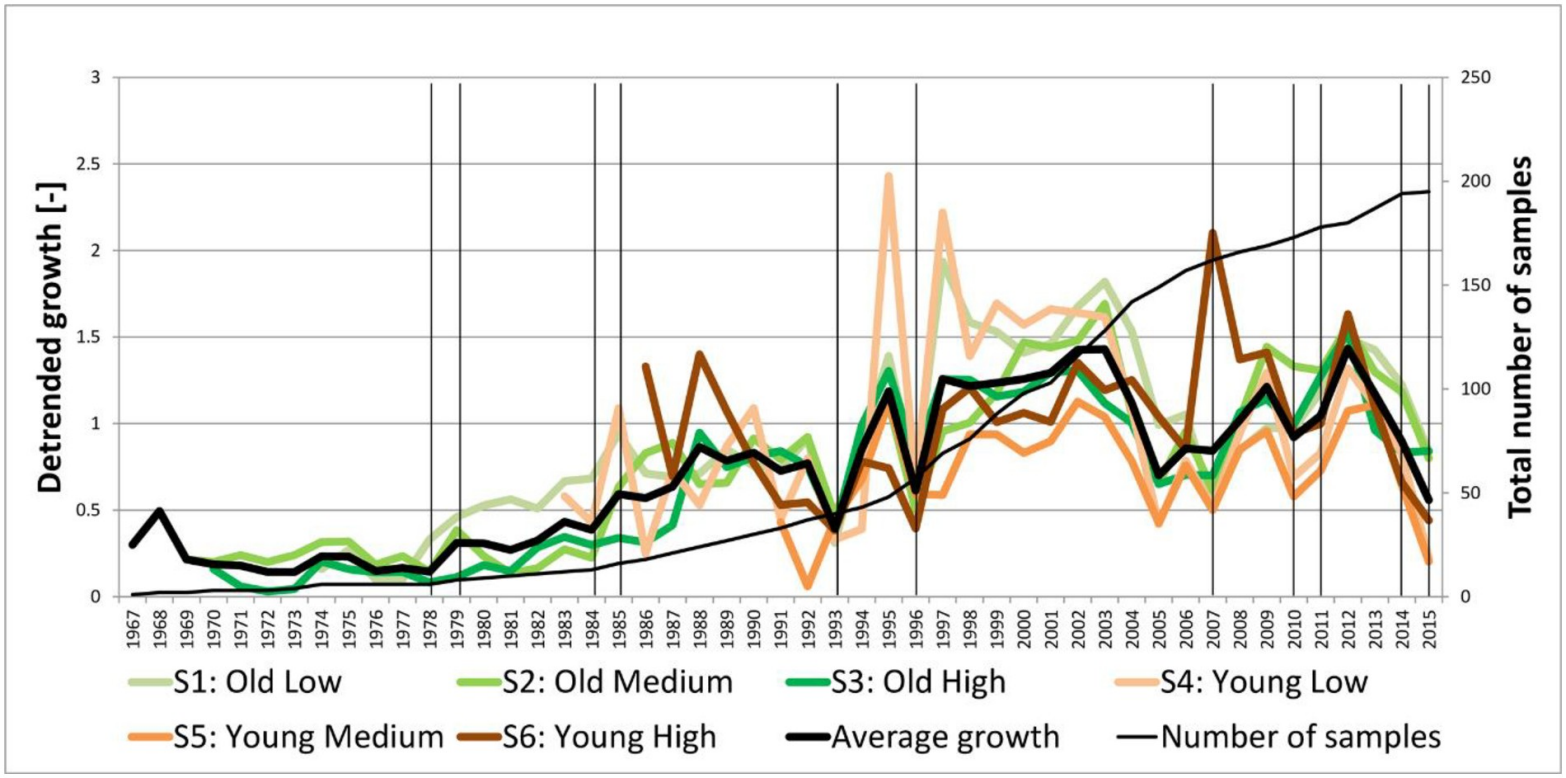
Average detrended ring-width chronologies for each stratum. The vertical lines indicate the occurrence of insect outbreaks by the brown-tail moth.

Average radial growth (based on raw ring-width series) over all years and strata was about 0.75 mm per year (all ring-width information can be found in S2 Table). Growth varied considerably from year to year, with a higher annual variation in shrubs growing on younger dunes and in lower areas (S4) than those in the older and higher areas (S3) (Fig 8). Despite the annual fluctuations in the younger dunes, average growth did not differ significantly between strata (ANOVA: F_5, 190_ = 1.889, P = 0.098). Year-to-year variation in growth was similar for all strata and indicates strong common responses to external factors that reduced growth in most of the sampled shrubs (e.g. 1993, 1996, 2005, 2007, 2010 and 2015). There was one major exception: in 2007, the time-series of stratum 6 (S6), the young dunes at high elevation, shows an opposite growth response (high average growth) in comparison to other strata. Sea-buckthorn shrubs in the low elevation strata tend to have more erratic growth and growth was generally lower in the young dunes compared to the old dunes in years where growth was reduced. A consistently higher growth rate was observed from 1997–2004. While de-trending removed the average age related growth trend, the oldest samples (> 35 years) tended to show low average growth early in their life-history (Fig 8). Trends in growth in the different strata over the period 1991–2015 were not significant (Linear regression: all strata, est. = 0.61, p = 0.46; S1, est. = 0.44, p = 0.71; S2, est. = 1.72, p = 0.066; S3, est. = 0.469, p = 0.54; S4, est. = -1.28, p = 0.45; S5, est. = 0.70, p = 0.39; S6, est. = 2.1, p = 0.06).

### Growth in relation to environmental variables

The results of the growth analysis in the longer period from 1975 to 2015 shows no significant effect of sea level on shrub growth for lower strata (Table 1: S1, est. = -4.6; S4, est. = -2.4; S5, est. = -2.6). However, moth outbreaks seem to be the strongest predictor for reduced growth in all strata (Table 1). The influence of groundwater is stronger in the strata at higher elevation (S3-S6), where a lower groundwater table can lead to drought stress. In general, temperature had a positive effect on sea-buckthorn growth (Table 1).

**Table 1:** Summarized results of multi-model inference over two periods: 1975–2015 and 1991–2015.

Period	Long period analysis						Short period analysis					
Years	1975>	1975>	1975>	1983>	1991>	1986>	1991>	1991>	1991>	1991>	1991>	1991>
Strata	S1	S2	S3	S4	S5	S6	S1	S2	S3	S4	S5	S6
**Intercept**	95***	83.6***	71.8*	106.7***	63.9***	100.9***	129.2***	97.9***	108***	127.3***	63.9***	95.8***
**Moth outbreaks**	-21.7*	-18.7*	-21.7***	-51.7*	-28.8*	-29.4*	-33.9		-30.5***	-68.8**	-28.8**	-26.9
**Temperature (GS)**	32.2**	30.7***	13.8	23.4		6.1	2.3	21.7	8.2			3.6
**Temperature (W)**		-19.2*	1.1		-9.2	2.1	4.94	-7.5	2.5		-9.2	
**Net precipitation (GS)**		5.9	6.5	-0.9		-2.4			10.2			
**Net precipitation (W)**			2.8		-4.7						-4.7	-6.4
**Groundwater (GS)**			-7.5		-32.6*	-44.5**	-2.7		-5.7	-16.6	-32.6*	-57.3*
**Groundwater (GS)**^**2**^	-12.8	-17.2**	0.8			-17.5	-39.4	-55.4**				-39.6
**Sea water floods**	-4.6			-2.4	-2.6		-21.3	-2.4		-27.5	-2.6	
**Moth x Groundwater**^**2**^						60.9	9.9					95.5
**Moth x Groundwater**						-7.7						-6
**Moth x Net precipitation (GS)**				19.5								
**Moth x Temperature (GS)**	-9.9		-13.2									
**Moth x Sea water floods**	-4.8											
**Net precipitation x Temperature (GS)**		-5.7		-20.6		10			-3.8			
**R**^2^	0.32	0.35	0.18	0.28	0.46	0.6	0.48	0.39	0.22	0.68	0.46	0.42
**Aw**	0.13	0.21	0.3	0.19	0.23	0.34	0.34	0.23	0.36	0.19	0.23	0.35

Significance levels are indicated with an asterisk (p < 0.05*; p < 0.01**; p < 0.001***). The model fit is shown at the bottom with the coefficient of determination (R2) and the Akaike weight (Aw). Sixteen variables were used in the analysis, with distinctions between growing season (GS) and winter (W). Several interactions (x) were also tested, but not all were included as they had no significant influence. Note that in the high elevation strata (S3 and S6) the seawater related variables were not included.

## Discussion

### Changes in sea-buckthorn cover due to succession

Before 1959 sea-buckthorn was mainly growing in higher areas of old dunes. In the period between 1959 and 1986 the most noticeable successional change was the increase in sea-buckthorn cover in the lower dune areas formed after 1950. The newly formed growing foredunes reduced sand dynamics and seawater influence in the areas behind the dunes, which favored the establishment of sea-buckthorn in these low areas. Between 1986 and 2000 the majority of the sea-buckthorn population was found in the younger dunes and was strongly reduced in the older dunes. It took the shrub roughly 30 years to become the dominant species in these dune areas spreading in a north to north-westerly direction over time. A similar time span needed for sea-buckthorn expansion into newly available habitat was reported for the islands Schiermonnikoog and Voorne [28,53]. On Ameland we found an overall increase in surface cover of sea-buckthorn over time, with the strongest increase in 2000–2009, especially in the younger dunes (Fig 5). Since sea-buckthorn is a nitrogen-fixing pioneer species it has the ability to colonize marginal soils, as is the case for the newly formed dunes. However, the *Hippophae* cover decreased dramatically in both young and old dunes between 2009 and 2014 (Fig 5). The drop in sea-buckthorn cover in the older dunes (especially at the lower and medium elevation dunes between 1986 and 2014) could be due to the ageing and replacement by other species such as *Sambucus nigra* L., *Crataegus monogyna* Jacq., *Rosa canina* L. and *Rubus fruticosus* L. [11, 28, 53,54]. The succession may be the result of competition for light, or unfavourable conditions created by nematodes (*Longidorus* sp.), which have been shown to increase at later successional stages of dune formation [11]. Nematodes reduce the vitality of the root system by limiting the phosphate uptake, hampering the plants’ nodulation capacity and causing a reduction of the nitrogen content [11]. By 2014 sea-buckthorn in the young dunes are not that young anymore (dune age is around 28 years). This means that even on the younger dunes some sea-buckthorn shrubs would have potentially reached an age which is 10 years older than the average shrub age, i.e. 18 years that was found in the entire studied population. Therefore it is plausible that also in the younger dunes the replacement of sea-buckthorn by other plant species was already ongoing since 2009 (S15 Fig., personal observations, [55]). Nevertheless, the drop in establishment within a time span of five years is quite sudden and could be—at least partially—related to unfavourable environmental factors (i.e. higher groundwater levels at lower elevation and more frequent insect outbreaks).

### Effects of environmental variables on sea-buckthorn growth

The results show that in most strata (both in the long and short time-series) outbreaks of brown-tail moths have a strong negative effect on growth. When looking at the growth curves we see that the low peaks in growth frequently coincide with the reported insect outbreaks: 1978, 1984, 1993, 1996, 2007, 2010–2011, 2014–2015. Also the frequency of the insect outbreaks has increased in the last decade, which is in line with the results of Moraal et al. (2011) [22] who found a shift in the frequency of insect outbreaks from the south to the north of the Netherlands. These moths cause partial to complete defoliation lowering the vitality of sea-buckthorn and creating more opportunities (e.g. more light) for other species to take over. While the sea-buckthorn population might recover from infrequent insect outbreaks, a further increase in frequency might also impact the severity and extent of the damage causing large scale mortality [15,56].

Seawater floods have increased since 1989 (S2 Fig), especially in lower areas. This is probably due to a combination of soil subsidence and an increase in storm occurrences and resultant flooding, which are triggered by dynamics in the North Atlantic Oscillation (NAO) [57]. However, we did not find any significant impact of seawater floods on the growth of sea-buckthorn. This could be related to the timing and duration of flooding events. Most floods occur in winter (S3 Fig), a period in which the resistance of shrubs is higher due to the fact that the cambium is dormant [58]. Only in 2015, seawater flooding occurred during the growing season, but its effect on growth is hard to disentangle from the insect outbreak that occurred in the same year. Since in all strata (both low and high elevation) a strong reduction in ring width is observed, it is more likely that defoliation by the brown-tail moth is the cause (Figs 7 and 8). Winter floods can affect the growth when the duration of the inundation is long and potentially lasts until the growing season. Sea-buckthorn roots will suffer due to asphyxiation which is also observed in other shrub species [7,29]. Slim (1997) [10] indicated that mortality of sea-buckthorn patches between 1989 and 1992 could be related to such seawater floods. Since we do not have long-term data on the duration of the inundation, groundwater levels (with multiple measurements per year) are a good indicator of the wetness during the growing season (S4 Fig). We found that the squared groundwater depth seems representative of saturated soils in the low and medium elevation old dunes. The growth of sea-buckthorn in those dunes was negatively affected by high groundwater levels and could indicate flooding stress (i.e. saturated soils) [7]. Especially in 2007, the flood frequency led to high groundwater levels (i.e. negative values) indicating potential flooding stress. Although an insect outbreak was reported, unlike 2015 the growth in the high elevation young dunes do not seem to be affected indicating that flooding stress has an effect on the growth reduction in the lower elevation strata. In the high elevation old dunes, growth did not remarkably differ from the previous years (at least not since 2005). In other years the effect of floods probably caused short inundation events that did not affect sea-buckthorn vitality. Most of the floods, e.g. between 1989 and 1994, did not lead to higher groundwater levels and values were even positive (i.e. dry conditions) for several years. Together with the relatively low net precipitation levels this could even have caused drought stress for the shrubs as indicated by the lower growth rates between 1989 and 1994. A possible lack of water supply is supported by the significant negative values for groundwater (GW) in the high and medium elevation young dunes (S5 and S6 –Table 1). Net precipitation itself did not significantly affect the growth of sea-buckthorn, but we did not specifically take into account drought periods. Also, we did not find significant interaction effects (e.g. groundwater x net precipitation), likely due to the complexity and interplay of different climate factors on growth. For interpretation of the results it has to be taken into account that ring-width data can be affected by multiple stressors prevailing during and before the growing season. Timing, duration and severity of short-term events largely determine the effect on shrub physiology and growth [59].

The subsidence and subsequent higher flooding frequencies also indirectly impact the sea-buckthorn vitality due to alterations in soil characteristics. More frequent flooding effects lead to increased salinity and saturation of the soil which are detrimental for the sea-buckthorn population [22,25].

If soil subsidence continues, and the frequency and duration of seawater flooding increases, our results indicate we can expect a further and accelerated decrease in sea-buckthorn cover, and according to Hoggart et al. (2013) [60], species richness in general [60]. Soil subsidence and sea-level rise might not only directly affect the growth of sea-buckthorn through flooding and/or high groundwater levels, but indirectly through changes in soil characteristics.

### Opportunities and shortcomings of combining data sources

Combining different data sources and methods, such as aerial photography and dendrochronology–as demonstrated in this study, proved to be successful for unravelling the population dynamics of sea-buckthorn. Both methods have their shortcomings: aerial photography provides discrete time series with varying quality. It is moreover sensitive to misclassifications due to species mixtures, and spectral overlap is unavoidable [32]. Dendrochronology relies on information gained from the actual vegetation and hence does not account for past mortality; i.e. graphs on population dynamics (Fig 7) do not include early established shrubs (e.g. 1960–1980) that have already died. However integrating both methods to study succession, population dynamics and environmental-growth relationships with high temporal resolution allows to make predictions on where sea-buckthorn will decline or thrive.

## Conclusions

The population dynamics of sea-buckthorn on Eastern-Ameland are related to landscape scale dune succession processes and fluctuations in environmental variables strongly linked to climate change such as the increase in sea-level rise and the occurrence of insect outbreaks. We found that, besides succession, brown-tail moths heavily impacted the vitality of sea-buckthorn and that the increased frequency of outbreaks likely accelerated the decrease of the sea-buckthorn population. Future sea-level rise will cause more frequent flooding, including during the growing season periods. A higher flood frequency will affect salinity of the soil and higher groundwater levels will cause saturated (anoxic) soil conditions. A higher groundwater level may lead to a decline of sea-buckthorn at low elevation, but might benefit growth at higher elevations by reducing the risk of drought stress. The expansion of sea-buckthorn towards higher elevations might negatively affect the biodiversity in the grey dunes habitat. Although the study shows evidence that the above mentioned factors have a clear effect on sea-buckthorn population dynamics, the interplay between factors is not always clear. This is particularly the case in 2015, where besides the ongoing natural succession, the effects of flooding and an insect outbreak could not be disentangled.

## Supporting information

S1 Table Aerial photograph metadata. (PDF)

S2 Table Descriptive statistics for the sampled shrubs in the six strata. The effect on the total time series of different steps in the correction process is also shown.
(PDF)

S1 Fig Annual growth vs age for all samples, with RCS detrending curve. (PDF)

S2 Fig Average flood frequency in different strata within the study area before and during the growing season. The frequency of flooding has increased since the start of gas extraction.
(PDF)

S3 Fig Seasonality of seawater floods, showing that flooding occurs predominantly in winter. The first flood events in April and July occurred in 2015.
(PDF)

S4 Fig Groundwater depth (cm) in the growing season (April-August). (PDF)

S5 Fig Net precipitation time-series for the whole year and growing, winter season. (PDF)

S6 Fig Mean temperatures time-series for the whole year and growing, winter season. (PDF)

S7 Fig Photograph taken in 1959 with green areas classified as sea-buckthorn. (PDF)

S8 Fig Photograph taken in 1986 with green areas classified as sea-buckthorn. (PDF)

S9 Fig Photograph taken in 2000 with green areas classified as sea-buckthorn. (PDF)

S10 Fig Photograph taken in 2009 with green areas classified as sea-buckthorn. (PDF)

S11 Fig Photograph taken in 2014 with green areas classified as sea-buckthorn. (PDF)

S12 Fig Sea-buckthorn cover changes between 1959 and 1986 (red = disappeared, yellow = appeared, blue = remained). (PDF)

S13 Fig Sea-buckthorn cover changes between 1986 and 2000 (red = disappeared, yellow = appeared, blue = remained). (PDF)

S14 Fig Sea-buckthorn cover changes between 2000 and 2009 (red = disappeared, yellow = appeared, blue = remained). (PDF)

S15 Fig Sea-buckthorn cover changes between 2009 and 2014 (red = disappeared, yellow = appeared, blue = remained). (PDF)

S16 Fig Sea-buckthorn cover changes between 1959 and 2014—Summed data over all strata. (PDF)
