# Supplementary material for: Population dynamics of Hippophae rhamnoides shrub in response of sea-level rise and insect outbreaks
Source: PLoS One. 2020 May 21;15(5):e0233011. doi: 10.1371/journal.pone.0233011 (PMC7242017; doi:10.1371/journal.pone.0233011)
Supplement: S1 Table — (PDF) [file pone.0233011.s001.pdf]

S1 Table. Aerial photograph metadata.

Year	Source	Scale	Type	Usage
2014	Topographic service Cadastre	1:18000	Colour	Yes
2011	Topographic service Cadastre	1:18000	Colour	No, heterogeneous image quality
2011	Topographic service Cadastre	1:18000	Colour	No, heterogeneous image quality
2009	Topographic service Cadastre	1:18000	Colour	Yes
2009	Topographic service Cadastre	1:18000	Colour	Yes
2004	Topographic service Cadastre	1:18000	Panchromatic	No, does not cover study area
2000	Archive	1:18000	Panchromatic	Yes
1996	Cadastre	1:18000	Panchromatic	No, insufficient quality
1992	Archive	1:18000	Panchromatic	No, does not cover study area
1990	Cadastre	1:18000	Panchromatic	No, insufficient quality
1986	Archive	1:18000	Panchromatic	Yes
1986	Archive	1:18000	Panchromatic	Yes
1979	Cadastre	1:18000	Panchromatic	No, insufficient quality
1969	Archive	1:20000	Panchromatic	No, insufficient quality
1959	Archive	1:20000	Panchromatic	Yes
1949	Cadastre	1:20000	Panchromatic	No, insufficient quality
