# Supplementary material for: Population dynamics of Hippophae rhamnoides shrub in response of sea-level rise and insect outbreaks
Source: PLoS One. 2020 May 21;15(5):e0233011. doi: 10.1371/journal.pone.0233011 (PMC7242017; doi:10.1371/journal.pone.0233011)
Supplement: S2 Table — The effect on the total time series of different steps in the correction process is also shown. (PDF) [file pone.0233011.s002.pdf]

S2 Table. Descriptive statistics for the sampled shrubs in the six strata. The effect on the total time series of different steps in the correction process is also shown.

General info & Stratum	Number of samples	Measured age	Establishment		Growth statistics					
		Mean age	*First*	*Mean*	*Mean*	*Median*	*Stdev*	*Skew*	*Gini*	*AR1*
Raw ring-width	196	18	1967	1999	0.75 mm	0.67 mm	0.48 mm	0.57 [-]	0.33%	0.28 [-]
Detrended ring-width	196	18	1967	1999	1.04 [-]	0.94 [-]	0.62 [-]	0.52 [-]	0.32%	0.26 [-]
1: Old Low	33	19	1974	1999	1.16 [-]	1.07 [-]	0.66 [-]	0.38 [-]	0.29%	0.25 [-]
2: Old Medium	33	22	1967	1996	1.13 [-]	1.02 [-]	0.60 [-]	0.46 [-]	0.30%	0.34 [-]
3: Old High	33	20	1970	1999	1.07 [-]	0.94 [-]	0.62 [-]	0.57 [-]	0.30%	0.25 [-]
4: Young Low	32	15	1983	2003	0.97 [-]	0.89 [-]	0.67 [-]	0.49 [-]	0.34%	0.19 [-]
5: Young Medium	32	15	1991	2001	0.79 [-]	0.74 [-]	0.51 [-]	0.44 [-]	0.35%	0.25 [-]
6: Young High	33	17	1986	2000	1.10 [-]	0.98 [-]	0.67 [-]	0.76 [-]	0.32%	0.31 [-]
