# Supplementary figures and images for: Population dynamics of Hippophae rhamnoides shrub in response of sea-level rise and insect outbreaks
Source: PLoS One. 2020 May 21;15(5):e0233011. doi: 10.1371/journal.pone.0233011 (PMC7242017; doi:10.1371/journal.pone.0233011)

**S1 Fig. Annual growth vs age for all samples, with RCS detrending curve.**

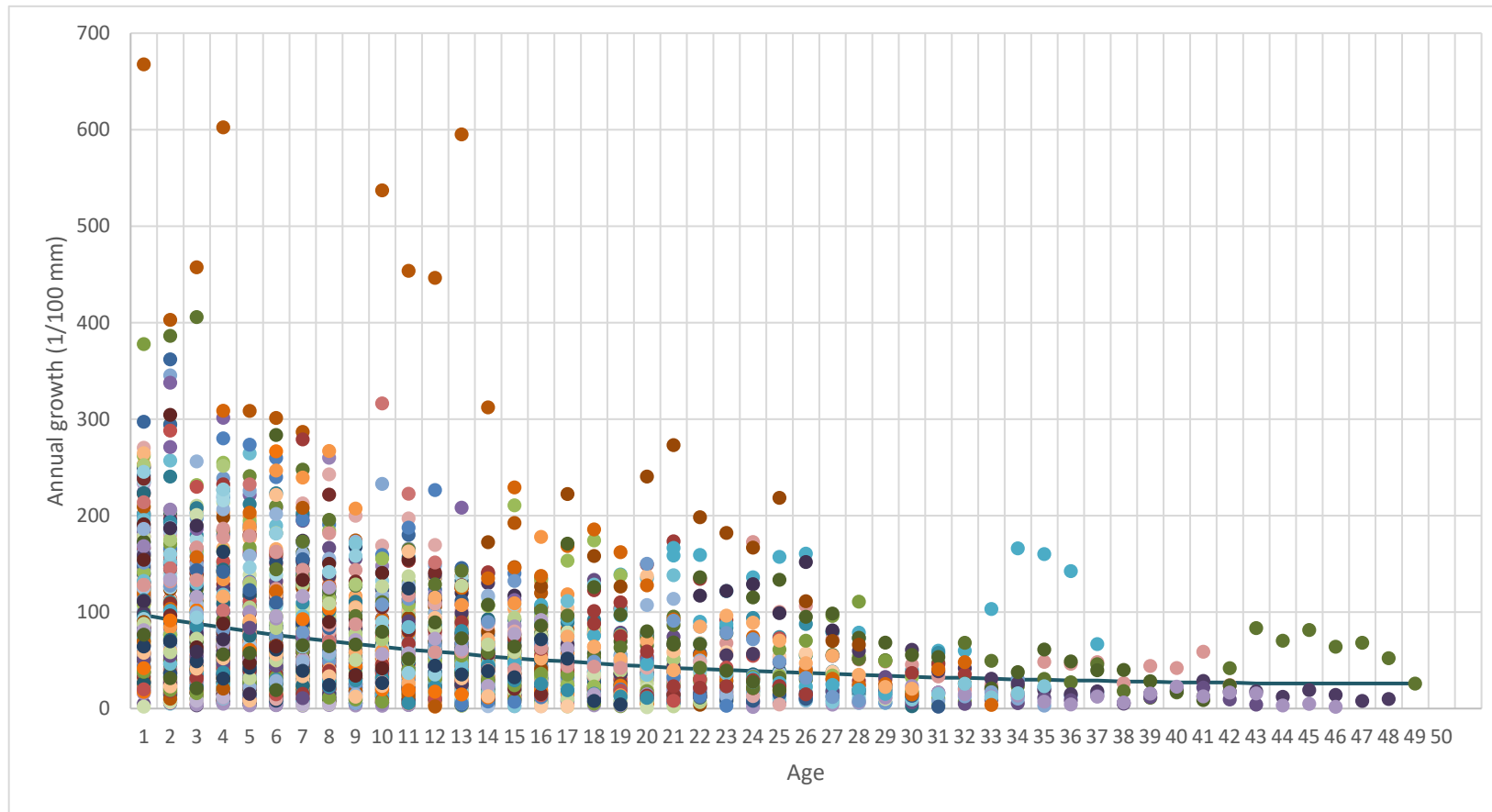

3

4

Supplement: S1 Fig — (PDF) [file pone.0233011.s003.pdf]

13 **S4 Fig. Groundwater depth (cm) in the growing season (April-August).**

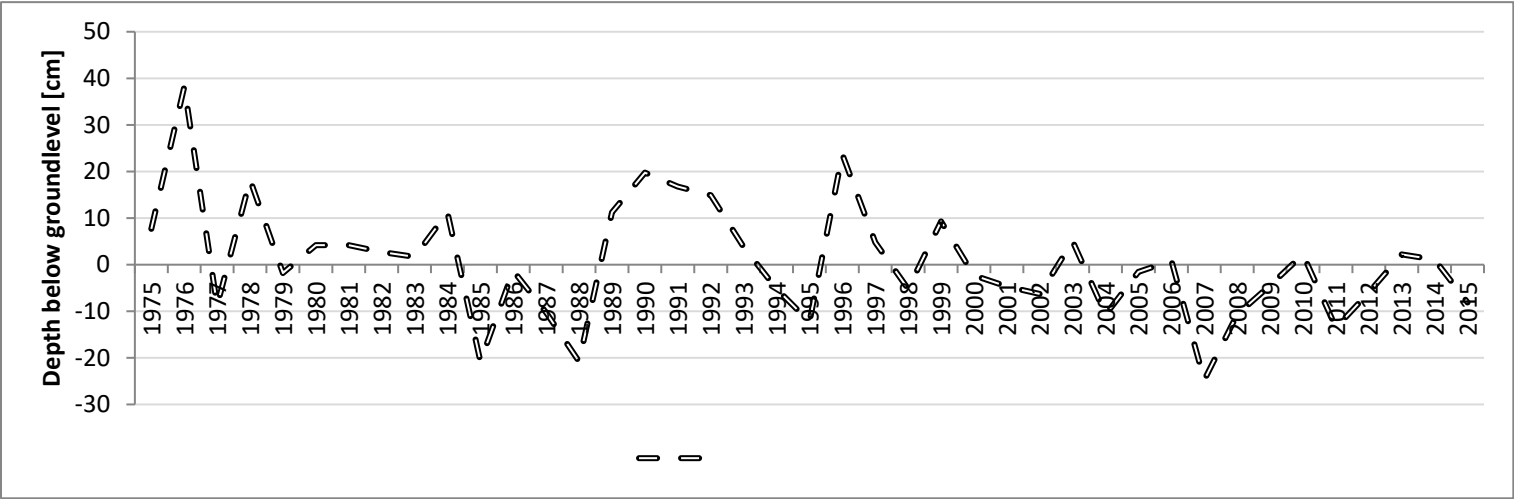

Supplement: S4 Fig — (PDF) [file pone.0233011.s006.pdf]

**S5 Fig. Net precipitation time-series for the whole year and growing, winter season**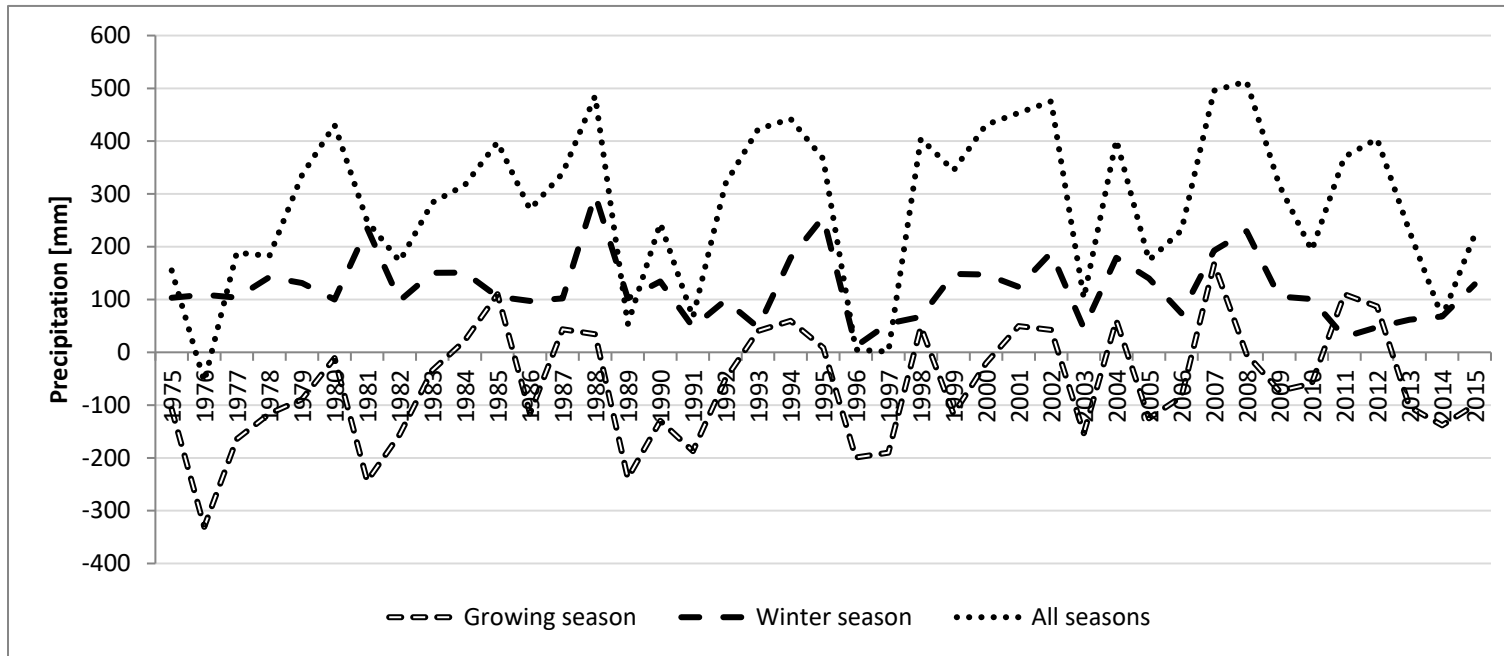

Supplement: S5 Fig — (PDF) [file pone.0233011.s007.pdf]

**S6 Fig. Mean temperatures time-series for the whole year and growing, winter season.**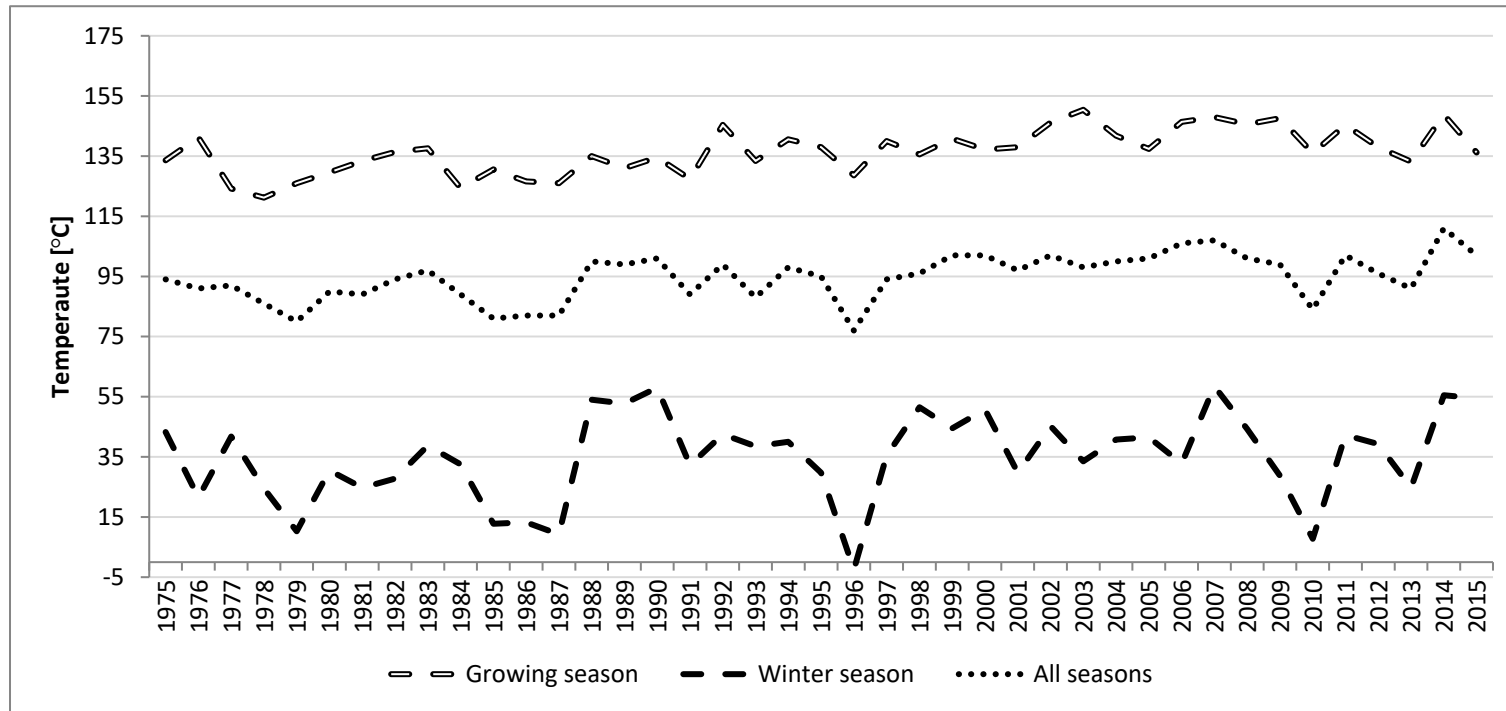

Supplement: S6 Fig — (PDF) [file pone.0233011.s008.pdf]

16 **S7 Fig. Photograph taken in 1959 with green areas classified**  
17 **as sea-buckthorn.**

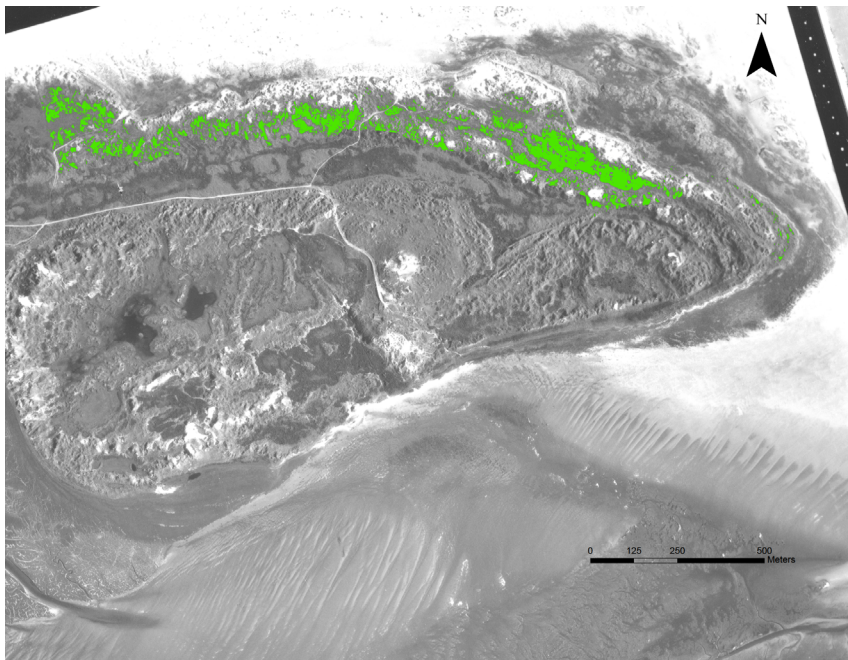

18

19

Supplement: S7 Fig — (PDF) [file pone.0233011.s009.pdf]

20 **S8 Fig. Photograph taken in 1986 with green areas classified**  
21 **as sea-buckthorn.**

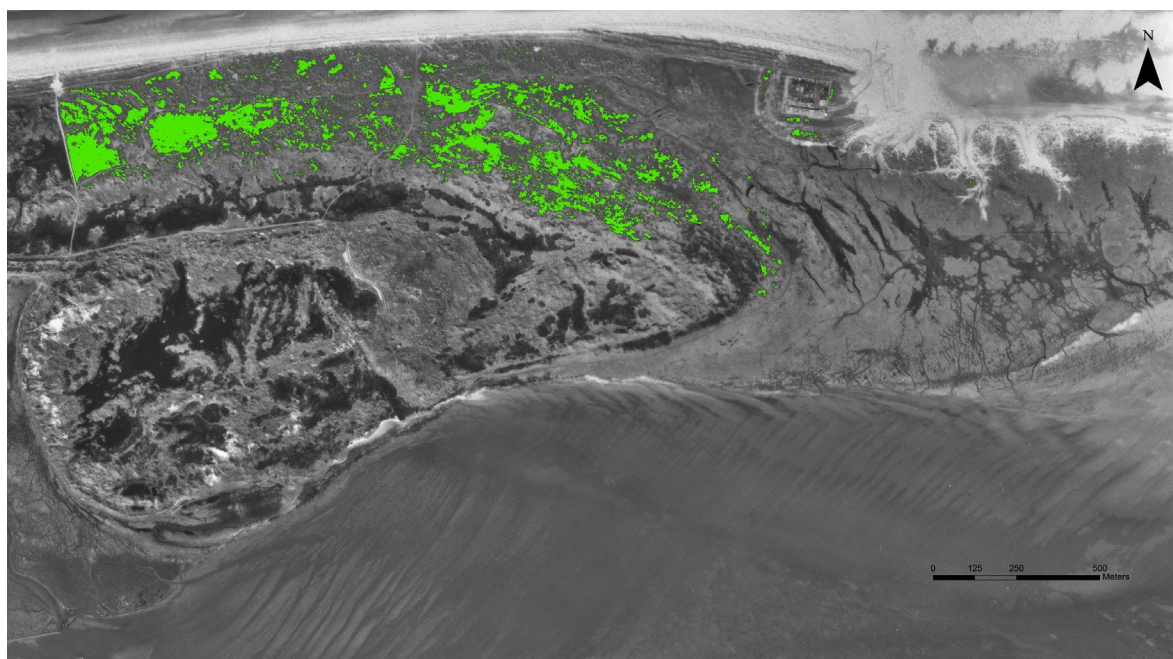

Supplement: S8 Fig — (PDF) [file pone.0233011.s010.pdf]

22

23 **S9 Fig. Photograph taken in 2000 with green areas classified**  
24 **as sea-buckthorn.**

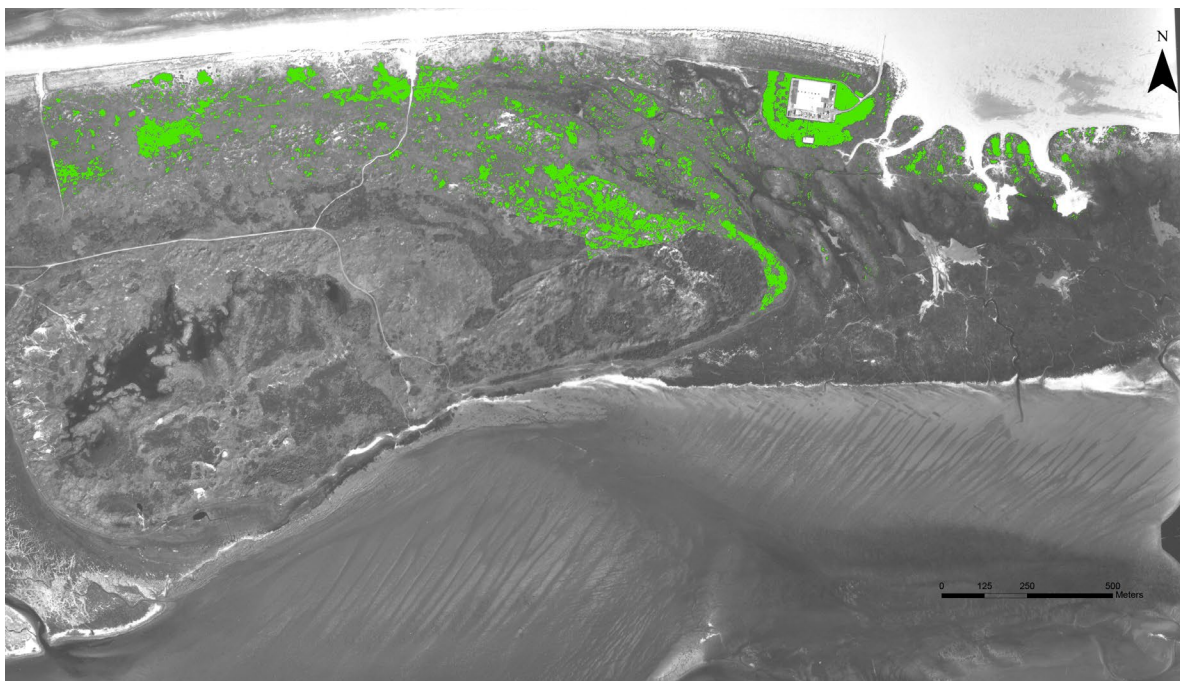

25

26

Supplement: S9 Fig — (PDF) [file pone.0233011.s011.pdf]

- 27 **S10 Fig. Photograph taken in 2009 with green areas**  
28 **classified as sea-buckthorn.**

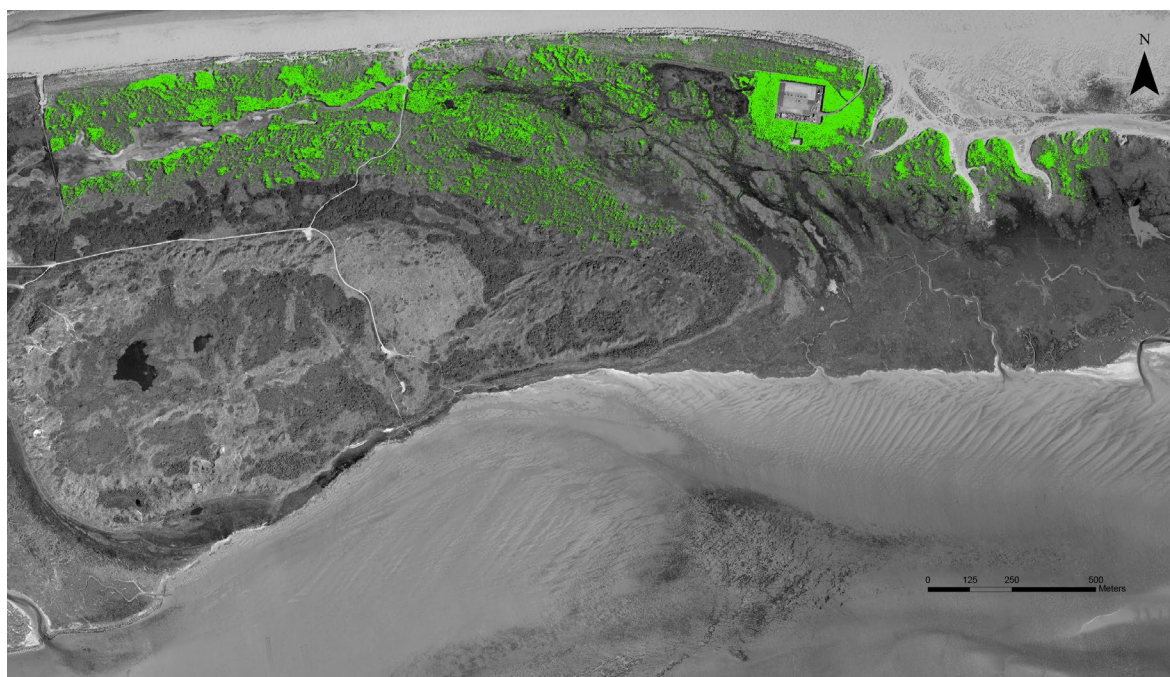

Supplement: S10 Fig — (PDF) [file pone.0233011.s012.pdf]

29

30 **S11 Fig. Photograph taken in 2014 with green areas**  
31 **classified as sea-buckthorn.**

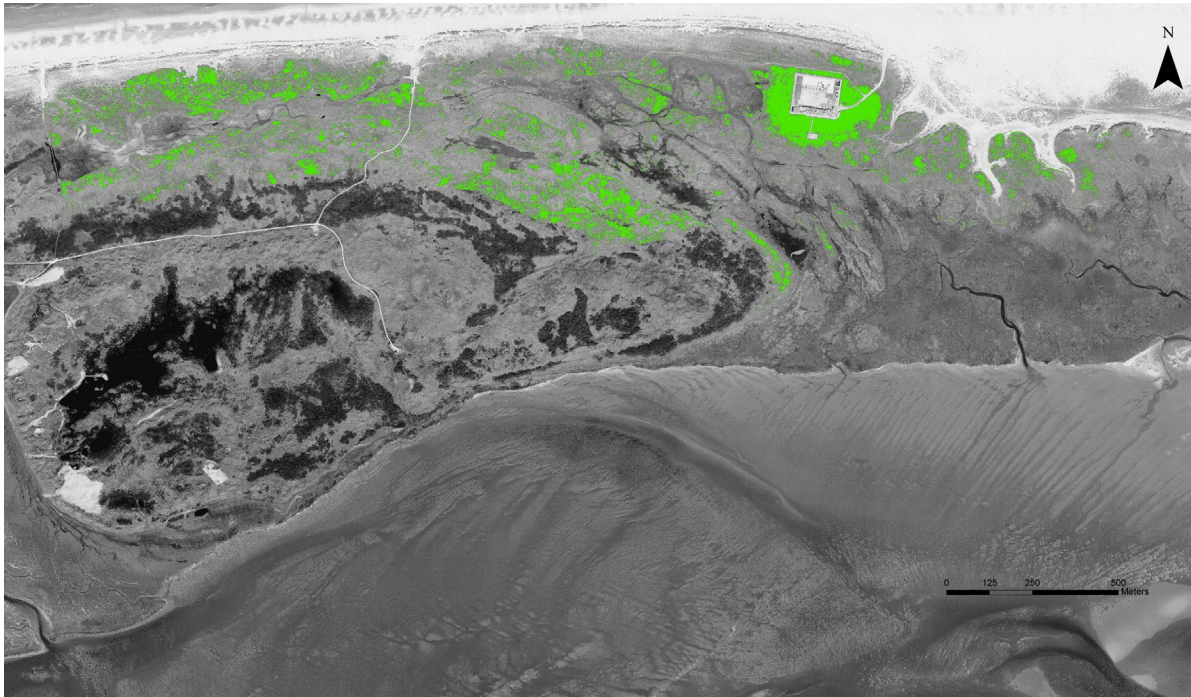

32

33

Supplement: S11 Fig — (PDF) [file pone.0233011.s013.pdf]

66 **S16 Fig. Sea-buckthorn cover changes between 1959 and**  
67 **2014 – summed data over all strata.**

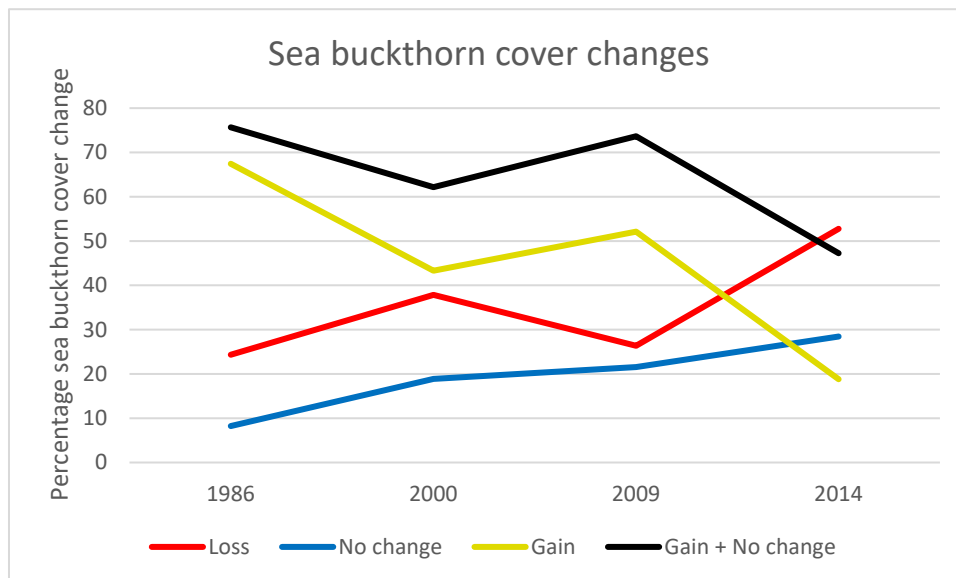

Supplement: S16 Fig — (PDF) [file pone.0233011.s018.pdf]
