# Supplementary material for: Population dynamics of Hippophae rhamnoides shrub in response of sea-level rise and insect outbreaks
Source: PLoS One. 2020 May 21;15(5):e0233011. doi: 10.1371/journal.pone.0233011 (PMC7242017; doi:10.1371/journal.pone.0233011)

S2 Fig. Average flood frequency in different strata within the study area before and during the growing season. The frequency of flooding has increased since the start of gas extraction in 1986.

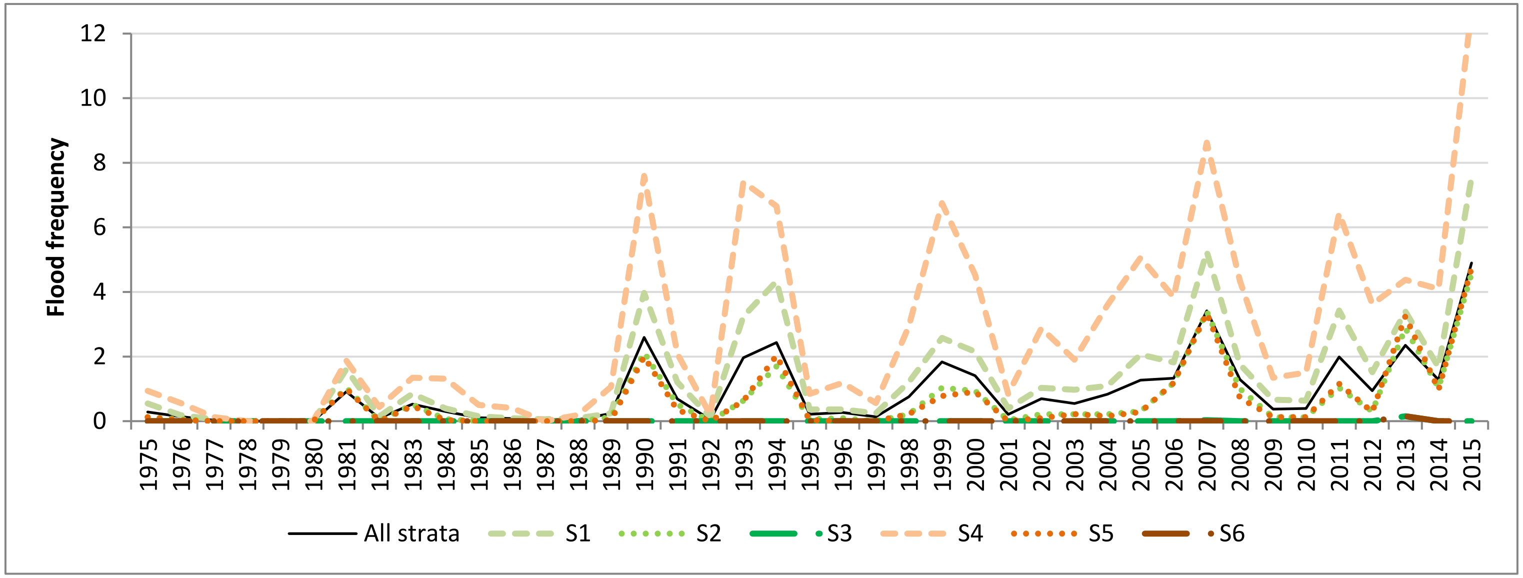

Supplement: S2 Fig — The frequency of flooding has increased since the start of gas extraction. (PDF) [file pone.0233011.s004.pdf]
