# Supplementary material for: Population dynamics of Hippophae rhamnoides shrub in response of sea-level rise and insect outbreaks
Source: PLoS One. 2020 May 21;15(5):e0233011. doi: 10.1371/journal.pone.0233011 (PMC7242017; doi:10.1371/journal.pone.0233011)

S3 Fig. Seasonality of seawater floods, showing that flooding occurs predominantly in winter. The first flood events in April and July occurred in 2015.

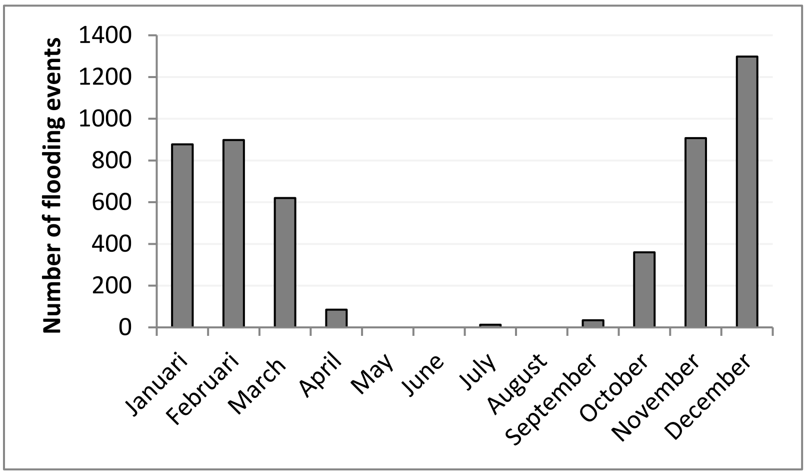

Supplement: S3 Fig — The first flood events in April and July occurred in 2015. (PDF) [file pone.0233011.s005.pdf]
