# Supplementary material for: Population dynamics of Hippophae rhamnoides shrub in response of sea-level rise and insect outbreaks
Source: PLoS One. 2020 May 21;15(5):e0233011. doi: 10.1371/journal.pone.0233011 (PMC7242017; doi:10.1371/journal.pone.0233011)

**S13 Fig. Sea-buckthorn cover changes between 1986 and 2000 (red=disappeared, yellow=appeared, blue=remained). The other colours represent the strata as denoted in Fig 2B of the article. Note that the larger blue/red patches in the far west are sea-buckthorn mixed with willow species (Salix sp.).**

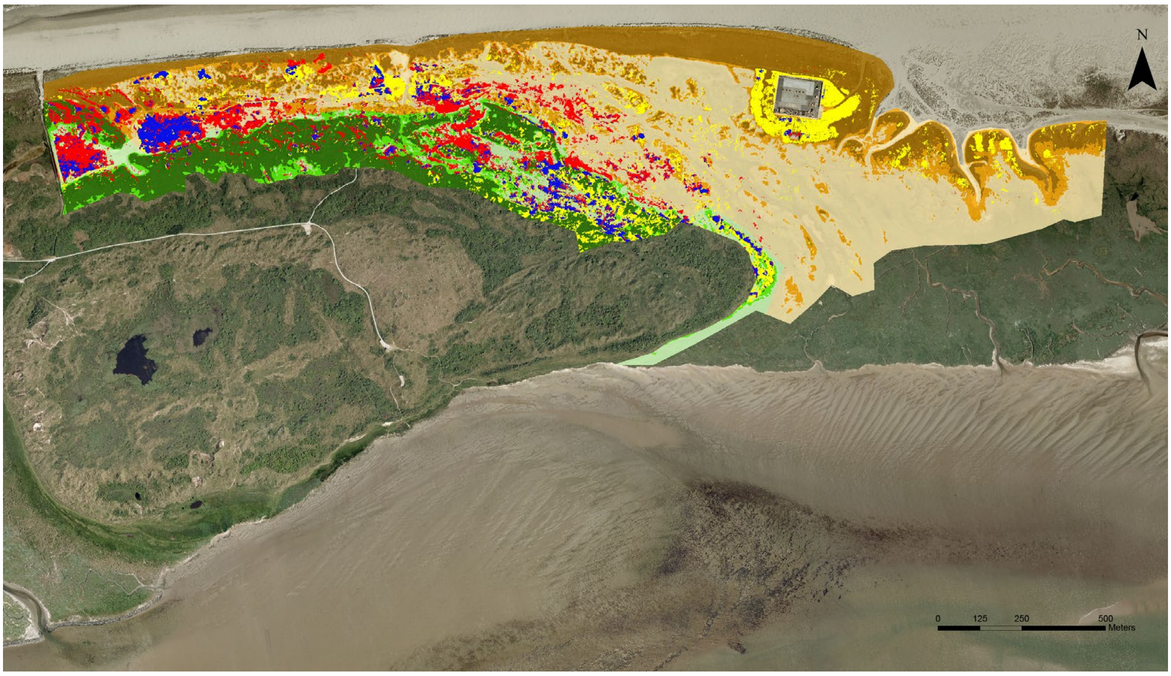

Supplement: S13 Fig — (PDF) [file pone.0233011.s015.pdf]
